# Root Canal Irrigants and Dentin Bonding: An Update

**DOI:** 10.22037/iej.2017.27

**Published:** 2017

**Authors:** Zahed Mohammadi, Shapour Yaripour, Sousan Shalavi, Flavio Palazzi, Saeed Asgary

**Affiliations:** a *Iranian Center for Endodontic Research, Research Institute of Dental Sciences, Dental School, Shahid Beheshti University of Medical Sciences, Tehran, Iran****; ***; b * Department of Oral and Maxillofacial Surgery, Hamedan University of Medical Sciences, Hamedan, Iran; *; c *Private Practice, Hamedan, Iran;*; d *Department of Neuroscience, Reproductive and Odontostomatological Sciences, Federico II University of Naples, Naples, Italy*

**Keywords:** Bond Strength, Chlorhexidine, EDTA, MTAD, Ozone, Sodium Hypochlorite

## Abstract

The purpose of the review was to assess the effect of root canal irrigants on dentin bonding. A PubMed-based search was conducted on the articles published from 1980 to 2016. A brief overview and reviewing the effect on dentin bonding of common root canal irrigation solutions such as sodium hypochlorite (NaOCl), chlorhexidine (CHX), ethylenediaminetetraacetic acid (EDTA), mixture of a tetracycline, acid and a detergent (MTAD) and ozone was conducted. Findings showed that, depending on the type of dentin bonding, using NaOCl may decrease, increase or not affect the bond strength. In addition, due to its broad-spectrum matrix metalloproteinase-inhibitory effect, CHX as well as MTAD can significantly improve the resin-dentin bond stability. However, the effect of ozone therapy on bond strength was controversial.

## Introduction

The conventional belief that all endodontically-treated teeth are weaker or more brittle than vital teeth, have led to the philosophy encouraging aggressive reinforcement of remaining tooth structure. Until recently, non-vital teeth were usually treated with a crown, core, and/or dowel which often led to remaining tooth structure being sacrificed for the preparation of a traditional cast restoration [1]. However, over-preparation of the post space and large diameter posts decrease the resistance against root fracture and increase the risk of apical pathosis [2]. The use of adhesive resin cement can compensate for the reduction of over-preparation for the post and dowel [1-4]. Endodontic therapy is a routine procedure for maintenance of non-vital teeth which consists of removing all contents of the root canal system during shaping. Successful cleaning entails the use of instruments to mechanically remove dentin, irrigants to flush loosened debris away and chemicals to dissolve contaminants from inaccessible regions [5]. Therefore, the purpose of this paper was to review the effects of root canal irrigants and medicaments on dentin bonding strength.

***Retrieval of literature***

An English-limited Medline search was performed through the articles published in PubMed from 1980 to 2016. The searched keywords included “Dentin Bonding AND Sodium Hypochlorite”, “Dentin Bonding AND Chlorhexidine", "Dentin Bonding AND MTAD”, “Dentin Bonding AND EDTA” and "Dentin Bonding AND Ozone". Then, a hand search was done in the references of result articles to find more matching papers. 

***Results***

A total of 927 articles were found which included “Dentin Bonding AND Sodium Hypochlorite” (309 articles), “Dentin Bonding AND Chlorhexidine" (250 articles), "Dentin Bonding AND MTAD” (20 articles), “Dentin Bonding AND EDTA” (328 articles) and "Dentin bonding AND Ozone" (20 articles). Of 927 searched documents, only English papers and full-text articles were used (book chapters and abstracts were not included). In addition, common search results between paired keywords were excluded. Therefore, of 927 searched documents, 80 were included in the study.

***Sodium hypochlorite***

Sodium hypochlorite (NaOCl) is recommended as the main endodontic irrigant because of its ability to dissolve organic matter together with its broad antimicrobial action [6]. NaOCl is commercially available as aqueous solutions with concentrations ranging from 1% to 15% and having an alkaline pH with values around 11 [6]. Among other salts, they also contain sodium hydroxide salts in order to increase their stability [7] and they might contain surfactants as well as other components that are not always disclosed by the manufacturer [7, 8]. 

***Effect on dentin bonding***

Dentin is degenerated by NaOCl due to dissolution of dentinal collagen [9]. Moreover, residual NaOCl may interfere with polymerization of bonding resin due to oxygen generation. The bond strength of resin following contact with NaOCl before etching decreases when a MMA-TBB resin system was employed [10]. The decreased bond strength is improved when neutralizing agents such as ascorbic acid or a sodium thiosulfate solution are applied after NaOCl treatment. These solutions remove NaOCl by the oxidation-reduction reaction. Nikaido *et al.* [11] evaluated the bonding strength at the buccal dentin surface after NaOCl treatment on the root canal wall dentin; the bonding strength of single bond (SB) significantly decreased after NaOCl treatment, while the bonding strength of self-etching primer system (Clearfil Mega Bond) did not change. Ishizuka *et al. *[9] found that while bonding strength of self-etching primer system decreased following NaOCl treatment that of SB did not change. Perdigao *et al.* [12] assessed the effect of 10% commercial NaOCl gel on the dentin shear bond strengths and hybrid layer ultra-morphology of two total-etch adhesive systems (Prime & Bond NT and Single Bond). Results demonstrated that the increase in the NaOCl application time resulted in a progressive decrease in shear bond strengths for both dentin adhesives. Frankenberger *et al.* [13] compared the dentin bond strength and marginal adaptation of direct composite resins with and without additional NaOCl treatment after the etching process. They found that after hypochlorite treatment, dentin bond strength and marginal adaptation decreased significantly. Saboia *et al.* [14] investigated the effect of 10% NaOCl for 1 min after acid conditioning on the shear bond strength of two acetone-based single-bottle adhesive systems and found that collagen removal improves the bond strength for these systems. Pioch *et al*. [15] evaluated the effect of NaOCl treatment of acid-etched dentin on the tensile bond strength of adhesive resins. They found that the removal of the collagen layer with NaOCl could enhance or decrease bond strengths, depending on the bonding agent used. Osorio *et al*. [16] evaluated the effect of NaOCl treatment on the shear bond strength and microleakage of a polyalkenoic acid-containing adhesive system. Results showed that adverse chemical interactions could have occurred between the remnant collagen matrix and/or mineralized dentin after NaOCl treatment. There was no additional advantage in using NaOCl treatment with this adhesive. Ari *et al*. [17] evaluated the effect of NaOCl on the regional bond strengths of four adhesive systems to root canal dentin. They found that, depending on the adhesive system, NaOCl enhanced the bond strength. Erdemir *et al*. [18] indicated that NaOCl, significantly decreased bond strength of C & B Metabond to root canal dentin. Shinohara *et al*. [19] found that depending on the adhesive system used, the application of NaOCl increased microleakage along dentin margins. Correr *et al*. [20] demonstrated that dentin surface treatment with NaOCl did not affect the resin-dentin bonding strength in primary teeth. Vongphan *et al*. [21] indicated that NaOCl significantly reduced the bond strengths of the adhesive when a total etching was applied. The application of sodium ascorbate on NaOCl treated dentin significantly improved the bond strengths. Wachlarowicz *et al*. [22] examined the effects of commonly employed endodontic irrigants on Epiphany-dentin bond strengths. They found that only NaOCl improved the bond strengths. Pucci *et al*. [23] evaluated the influence of collagen removal with 10% sodium hypochlorite (10% NaOCl) on the longitudinal shear bond strength (SBS) of adhesives to dentin.

***Chlorhexidine***

Chlorhexidine (CHX), a cationic bisguanide, is stable as a salt although it dissociates in water at a physiologic pH, releasing the CHX component [24]. It is frequently used at concentrations between 0.2% and 2% and exhibits an optimal antimicrobial activity at a pH of 5.5 to 7.0 depending on the buffering agent used and the under-study organism. The most common preparation is CHX gluconate [25]. It has been recommended that CHX can be used as either an alternative or an adjunct to root canal irrigant because of its antimicrobial qualities. Studies comparing its antimicrobial action versus NaOCl solutions present conflicting results [25, 26].

***Role of CHX in stabilizing the organic matrix of the resin-dentin bond***

During the last two decades, chemical and technical advances have contributed to increases in resin-dentin bond strength. However, the premature loss of bond strength is one of the problems that still affects adhesive restorations [27] and markedly reduces their durability [28-30]. The loss of bond strength has been attributed mainly to the degradation of the hybrid layer at the dentin-adhesive interface. Numerous publications have demonstrated this lack of bond stability [31-34]. Shortly, deterioration of dentin collagen fibrils contributes to the mechanisms responsible for bond degradation [35, 36]. In this context, it has been speculated that a decreasing concentration gradient of resin monomer diffusion within the acid-etched dentin, and a subsequent resin elution from hydrolytically unstable polymeric hydrogels within the hybrid layers leaves the collagen fibrils unprotected and vulnerable to degradation by endogenous matrix metallo-proteinase (MMPs) [31]. The MMPs are a group of 23 mammalian enzymes capable of degrading all extracellular matrix components. Human dentin contains at least collagenase (MMP-8), gelatinases MMP-2 and -9, and enamelysin MMP-20 [37-40]. Dentin collagenolytic and gelatinolytic activities can be suppressed by protease inhibitors [36], indicating that MMP inhibition could be beneficial in the preservation of hybrid layers. This was demonstrated in an *in vivo* study, in which the application of CHX, known to have a broad-spectrum MMP-inhibitory effect [41], significantly improved the integrity of the hybrid layer in a six-month clinical trial [42]. Carrilho *et al.* [43] evaluated the *in vitro* effect of CHX on the resin-dentin bond stability. Results showed that with CHX, significantly better preservation of bond strength was observed after 6 months and protease inhibitors in the storage medium had no effect. Failure analysis showed significantly less failure in the hybrid layer with CHX, compared with controls after 6 months. Furthermore, they evaluated the effect of CHX on the preservation of the hybrid layer *in vivo*. Findings showed that bond strength remained stable in the CHX-treated specimens, while bond strength decreased significantly in control teeth. Resin-infiltrated dentin in CHX-treated specimens exhibited normal structural integrity of the collagen network. Conversely, progressive disintegration of the fibrillar network was identified in control specimens. They concluded that auto-degradation of collagen matrices can occur in resin-infiltrated dentin, but may be prevented by the application of a synthetic protease inhibitor, such as CHX [44].

A recent study evaluated the effect of CHX application protocol on durability of marginal seal of class V composite restorations [45]. Findings showed that application of CHX after etching without rinsing is effective to decrease microleakage. However, it had no effect if applied before etching in use of this particular type of etch-and-rinses adhesive after thermocycling.

On the whole, due to its broad-spectrum MMP-inhibitory effect, CHX can significantly improve the stability of resin-dentin bond.

***MTAD***

Mixture of a tetracycline, acid and a detergent (BioPure MTAD) (Dentsply, Tulsa Dental, Tulsa, OK, USA), is a root canal irrigant introduced by Torabinejad *et al.* [46, 47]. The solution is a mixture of 3% doxycycline, 4.25% citric acid and a detergent (0.5% Polysorbate 80) [46-49]. Several studies have evaluated the effectiveness of MTAD for disinfection of root canals. Torabinejad *et al.* [46] have shown that MTAD is able to remove the smear layer and is effective against *E. faecalis*. Newberry *et al*. showed that MTAD inhibited growth of most strains of *E. faecalis* when diluted 1:8192 times and killed most strains of *E. faecalis* when diluted 1:512 times. Antibacterial efficacy of MTAD has been revealed in some other studies. 

Torabinejad *et al*. [46, 47] showed that MTAD effectively removed smear layer and did not significantly change the structure of the dentinal tubules when used after NaOCl (5.25%) as compared with EDTA and 5.25% NaOCl irrigation. Park *et al*. [49] sated that smear-layer removal using MTAD had no significant effect on decreasing the coronal leakage compared to EDTA. These findings were confirmed by Ghoddusi *et al.* [50].

Substantivity of MTAD has been demonstrated for up to 4 weeks [49, 50]. Furthermore, it has been indicated that MTAD is somewhat effective against bacterial biofilms, however, it cannot disrupt biofilms completely [51]. MTAD does not adversely affect the physical properties (*i.e.* flexural strength and modulus of elasticity) of dentin [52]. 

***Effect on dentin bonding***

Tetracyclines have also been shown to inhibit mammalian collagenases. Inflammatory diseases such as periodontitis include a pathological excess of tissue collagenases that may be blocked by tetracyclines, leading to enhanced formation of collagen and bone formation. Doxycycline, a hydroxyl derivative of tetracycline, is the most potent anticollagenase antibiotic among commercially available tetracyclines [53], and is also relatively more potent against most periodontal pathogens [53].

Machnick *et al*. [48] compared the effect of MTAD and phosphoric acid on the bond strength to enamel and dentin using a conventional OptiBond Solo Plus dentin adhesive system, and reported that teeth treated with MTAD protocol (20 min 1.3% NaOCl/5 min MTAD) might not need any additional dentin conditioning before the application of the dental adhesive. Garcia-Godoy *et al*. [54] evaluated the structure of the hybrid layer formed after the use of EDTA or MTAD solutions when used as a final flush. Findings showed that the BioPure MTAD hybrid layer was thicker than the 17% EDTA hybrid layer. Both the BioPure MTAD and EDTA caused a collapse of the dentin matrix structure, which impeded sealer infiltration and the formation of high quality hybrid layer bonding. The hybrid layers created in smear layer-covered dentin exhibited less potential for nanoleakage than the MTAD or EDTA hybrid layers. Wachlarowicz *et al*. [22] reported that neither EDTA nor MTAD significantly improved Epiphany-dentin bond strengths when compared with NaOCl used alone. Gopikrishna *et al.* [55] found that MTAD, as a final rinse, decreased the bond strength of AH-Plus and Apexit. Kandaswamy *et al*. [56] evaluated the effect of MTAD, EDTA and HEBP on the shear bond strength of AH-Plus sealer to coronal dentin. According to their findings EDTA showed highest bond strength followed by HEBP and MTAD. Mortazavi *et al.* [57] demonstrated that that the use of clinical protocol of 1.3% NaOCl as a root canal irrigant and a 5-min application of MTAD as a final rinse to remove the smear layer had no adverse effect on the shear bond strength of self-etch adhesives to dentin. 

On the whole, due to its broad-spectrum MMP-inhibitory effect, MTAD can significantly improve the resin-dentin bond stability. 

***EDTA***

Ethylenediaminetetraacetic acid (EDTA) is a chelating amino acid which is widely used to sequester di- and trivalent metal ions. EDTA binds to metals through four carboxylate and two amine groups. EDTA forms especially strong complexes with Mn, Cu, Fe and Co [56]. EDTA is a polyaminocarboxylic acid and a colorless, water-soluble solid. It is widely used to dissolve lime scale. Its usefulness arises because of its role as a hexadentate ligand and chelating agent which enables it to sequester metal ions such as Ca^2+^ and Fe^3+^ [57]. After being bound by EDTA, metal ions remain in solution, but exhibit diminished reactivity. EDTA is produced as several salts, notably disodium EDTA and calcium disodium EDTA. EDTA reacts with the calcium ions in dentin and forms soluble calcium chelates. It has been reported that EDTA decalcified dentin to a depth of 20-30 μm in 5 min [58].

Morris *et al.* [59] found that both NaOCl and EDTA significantly reduced the bond strength of resin cement to root dentin. Perdigao *et al.* [60] showed that this reduction can be completely reversed by application of 10% ascorbic acid or 10% sodium ascorbate. Michiels *et al*. [61] showed that the reduction in through and through leakage was significantly higher with the Nd:YAG laser as smear layer modifier than when smear layer is removed with an EDTA rinsing solution. Nunes *et al.* [62] showed that treating dentin with a combination of 1% NaOCl and 17% EDTA produced stronger adhesion of AH-Plus sealer compared to 1% NaOCl alone. 

***Ozone***

Ozone (O_3_) is a triatomic molecule consisting of three oxygen atoms. Its molecular weight is 47, 98 g/mol and thermo-dynamically highly instable compound that, depending on system conditions like temperature and pressure, decompose to pure oxygen with a short half-life [61]. Ozone is 1.6-fold denser and 10-fold more soluble in water (49.0 mL in 100 mL water at 0^°^C) than oxygen. Although ozone is not a radical molecule, it is the third most potent oxidant after fluorine and per sulfate. Ozone is an unstable gas that cannot be stored and should be used at once because it has a half-life of 40 min at 20^°^C [62]. Ozone (O_3_) is naturally produced by the photo dissociation of molecular oxygen (O_2_) into activated oxygen atoms, which then react with further oxygen molecules. This transient radical anion rapidly becomes protonated, generating hydrogen trioxide (HO_3_), which, in turn, decomposes to an even more powerful oxidant, the hydroxyl radical (OH) [61, 62]. It is the fundamental form of oxygen that occurs naturally as a result of ultraviolet energy or lightning, causing a temporary recombination of oxygen atoms into groups of three. In the clinical setting, an oxygen/ozone generator simulates lightning *via* an electrical discharge field. Ozone gas has a high oxidation potential and is 1.5 times greater than chloride when used as an antimicrobial agent against bacteria, viruses, fungi, and protozoa. It also has the capacity to stimulate blood circulation and the immune response. Such features justify the current interest in its application in medicine and dentistry and have been indicated for the treatment of 260 different pathologies [62].

***Effect on dentin bonding***

Schmidlin *et al.* [63] evaluated the influence of direct high-dose gaseous ozone application (2100 ppm) on dentin and enamel shear bond strength. According to their findings, despite a possible retention of surface and subsurface oxide-related substances during high-dose ozone application, shear bond strength was not impaired. Thus, adhesive restoration placement should be possible immediately after ozone application for cavity disinfection. Bitter *et al.* [64] showed that adhesion of the self-adhesive resin cement RelyX Unicem was significantly reduced after using gaseous ozone. 

Magni *et al.* [65] indicated that Ozone gas did not compromise the mechanical properties of the adhesives. Cadenaro *et al.* [66] demonstrated that the use of ozone gas to disinfect the cavity before restoration had no influence on immediate enamel and dentin bond strength. Çehreli *et al.* [67] revealed that pre-treatment with ozone improved the marginal sealing ability of the fissure sealants. Bojar *et al.* [68] showed that ozone therapy improved shear bond strength of two root canal sealers (AH-26 and EX Fill). Gurgan *et al.* [69] showed that ozone treatment did not impair the shear bond strength of two self-etch adhesives (Clearfil SE Bond and Clearfil Tri-S Bond) used for coronal and radicular dentin. According to Rodriguez *et al.* [68] ozone decreased the microtensile bond strength of dentin-composite resin interface. Dalkilic *et al.* [70] indicated that ozone reduced the initial microtensile bond strength of Clearfil SE Bond. According to Arslan *et al.* [71] ozone did not significantly affect the dentin bond strength of a silorane-based resin composite, filtek supreme. Garcia *et al.* [72] revealed that ozone gas and ozonated water used before the bonding process have no deleterious effects on the bond strengths and interfaces.

## Conclusion

NaOCl may decrease, increase or not affect the bond strength, depending on the type of bonding system. In addition, CHX and MTAD can significantly improve the resin-dentin bond stability which is attributed to their MMP-inhibitory effect. The effect of ozone therapy on bond strength was controversial.
